# Menadione-resistant Chinese hamster ovary cells have an increased capacity for glutathione synthesis.

**DOI:** 10.1038/bjc.1997.477

**Published:** 1997

**Authors:** K. A. Vallis, J. Reglinski, M. Garner, M. M. Bridgeman, C. R. Wolf

**Affiliations:** Imperial Cancer Research Fund Molecular Pharmacology Unit, Biomedical Research Centre, Ninewells Hospital and Medical School, Dundee, UK.

## Abstract

A cell line (MRc40) resistant to the model quinone compound, menadione, has been isolated from a parental Chinese hamster ovary cell line (CHO-K1). The known relationship between menadione toxicity and glutathione (GSH) depletion led us to investigate whether the mechanism of resistance of MRc40 was related to alteration in GSH homeostasis. Intracellular concentrations of GSH and cysteine (CySH) were twofold and 3.2-fold greater in MRc40 than in CHO-K1. Following exposure to menadione, GSH and CySH were depleted, but subsequent recovery of thiols was more rapid and of greater magnitude in MRc40 than in CHO-K1. Twelve hours after exposure to menadione, the concentrations of GSH and CySH were 9.7- and 4.2-fold greater in MRc40 than in CHO-K1. Using nuclear magnetic resonance (NMR) spectroscopy, we observed the in situ removal of menadione from cell suspensions of CHO-K1 and MRc40. However, only in CHO-K1 did we observe concomitant depletion of NMR-visible GSH. We conclude that the perturbation of GSH metabolism contributes to the resistant phenotype and is an important characteristic of menadione-resistant CHO cells.


British Journal of Cancer (1997) 76(7), 870–877
© 1997 Cancer Research Campaign

### Menadione-resistant Chinese hamster ovary cells have an increased capacity for glutathione synthesis

**KA Vallis[1]*, J Reglinski[2], M Garner[2], MME Bridgeman[3] and CR Wolf[1]**

[1]Imperial Cancer Research Fund Molecular Pharmacology Unit, Biomedical Research Centre, Ninewells Hospital and Medical School, Dundee DD1 9SY; [2]Department of Pure and Applied Chemistry, Strathclyde University, Glasgow G1 1XL; [3]Department of Biochemistry, University of Edinburgh, Edinburgh EH8 9XD, UK

**Summary** A cell line (MRc40) resistant to the model quinone compound, menadione, has been isolated from a parental Chinese hamster ovary cell line (CHO-K1). The known relationship between menadione toxicity and glutathione (GSH) depletion led us to investigate whether the mechanism of resistance of MRc40 was related to alteration in GSH homeostasis. Intracellular concentrations of GSH and cysteine (CySH) were twofold and 3.2-fold greater in MRc40 than in CHO-K1. Following exposure to menadione, GSH and CySH were depleted, but subsequent recovery of thiols was more rapid and of greater magnitude in MRc40 than in CHO-K1. Twelve hours after exposure to menadione, the concentrations of GSH and CySH were 9.7- and 4.2-fold greater in MRc40 than in CHO-K1. Using nuclear magnetic resonance (NMR) spectroscopy, we observed the in situ removal of menadione from cell suspensions of CHO-K1 and MRc40. However, only in CHO-K1 did we observe concomitant depletion of NMR-visible GSH. We conclude that the perturbation of GSH metabolism contributes to the resistant phenotype and is an important characteristic of menadione-resistant CHO cells.

**Keywords:** menadione; glutathione; Chinese hamster ovary; nuclear magnetic resonance; high-performance liquid chromatography

Menadione, 2-methyl-1,4-naphthoquinone, is a quinone that undergoes redox cycling with generation of a flux of superoxide anions (Mason, 1982). It has been investigated as a chemotherapeutic agent (Chlebowski et al, 1985) and has been used to sensitize tumours to other chemotherapeutic agents (Margolin et al, 1995). The closely related compound, 2-methyl-1,4-naphthoquinol-bis-disodium phosphate (synkavit), has been used to enhance the effect of ionizing radiation in patients being treated for cancer (Deeley, 1962). Menadione has also been used as a model compound to investigate the effects of oxidative stress in both prokaryotic and eukaryotic cells (Thor et al, 1982; Greenberg et al, 1990).

A one-electron reduction of menadione to its semiquinone radical is catalysed by flavoenzymes, including NADPH-cytochrome P-450 reductase. Quinone acceptor oxidoreductase (QAO) (formerly DT-diaphorase) reduces menadione by a two-electron transfer to its hydroquinone. Since the hydroquinone is relatively unreactive, the action of QAO is protective. The semiquinone radical reacts with molecular oxygen to form superoxide radicals and then, by dismutation, hydrogen peroxide. This interacts with metal ions to generate hydroxyl radicals. The semiquinone also reacts directly with glutathione (GSH) to form glutathione disulphide (GSSG) (Ross et al, 1985). Glutathione reductase catalyses the regeneration of GSH, which maintains the cell in a reduced state. Menadione also interacts with glutathione to form a menadione–GSH conjugate (Nickerson et al, 1963) and with protein-SH groups to form arylated protein (Di Monte et al, 1984).

*Received 5 November 1996*
*Revised 20 March 1997*
*Accepted 24 March 1997*

*Correspondence to:* CR Wolf

GSH plays a pivotal role in the protection of cells from the toxic effects of oxidants. Several cell lines that are resistant to chemical oxidants have elevated GSH (Wolf et al, 1987; Kramer et al, 1988). Elevation of intracellular GSH is also associated with increased resistance to ionizing radiation (Russo et al, 1985; Vos and Roos-Verhey, 1988). Conversely, lowering intracellular thiols using the inhibitor of glutathione synthesis, buthionine sulphoximine, sensitizes cells to oxidants (Dethmers and Meister, 1981). We have previously reported the isolation of menadione-resistant cell lines that overexpress GSH (Vallis and Wolf, 1996). Although elevation of GSH has been observed in cell lines resistant to oxidants, the mechanism underlying this increase has not been determined. In drug resistance, it is not only GSH concentration that determines sensitivity to drug but also the capacity of cells to resynthesize GSH in the presence of oxidant. We have made dynamic measurements using biochemical and nuclear magnetic resonance (NMR) techniques to evaluate changes in the capacity of menadione-resistant cells to synthesize GSH.

Spin-echo pulse sequence NMR takes advantage of the differences in relaxation times between large and small molecules in solution. It is possible to tune out signals arising from large molecules to produce a spectrum composed solely of small molecules, of which GSH is an important example. NMR has proved useful in the non-invasive measurement of thiol redox status (Livesey et al, 1992), as alteration of the GSH–GSSG ratio is easily visualized (McKay et al, 1986; Al-Kabban et al, 1988). Using this approach, it is possible to observe subtle changes in glutathione chemistry.

The purpose of this study was to investigate alteration in GSH homeostasis in a Chinese hamster ovary (CHO) cell line following acquisition of resistance to menadione. The complementary techniques of high-performance liquid chromatography (HPLC),

**Present address:* Ontario Cancer Institute/Princess Margaret Hospital, 610 University Avenue, Toronto, Ontario M5G 2M9, Canada which provides accurate quantitative data, and NMR, which provides a dynamic measure of glutathione, were used.

### MATERIALS AND METHODS

### Reagents

Menadione, menadione bisulphite, cysteine and glutathione were obtained from Sigma Chemical. Monobromobimane (mMBBr) was obtained from Calbiochem UK Cambridge. Glacial acetic acid, methanol and acetonitrile (all HPLC grade) were obtained from BDH. *N*-ethyl morpholine, dithiothreitol and $^2H_2O$ were obtained from Merck, Boehringer-Mannheim and Goss Scientific Instruments respectively.

### Cell culture

The cell line, MRc40, was isolated from a Chinese hamster ovary cell line (CHO-K1) (Vallis and Wolf, 1996). Menadione was added to logarithmically growing parental cells. The initial concentration of menadione was 20 μM. This was increased to 30 μM and finally to 40 μM as resistance developed. Cells were routinely maintained in α-minimal essential medium supplemented with 10% fetal calf serum, glutamine (2 mM), streptomycin (100 μg $ml^{-1}$) and penicillin (100 IU $ml^{-1}$). Cells were grown in monolayer at 37°C in a humidified atmosphere containing 5% carbon dioxide. The $IC_{50}$ for menadione was 7.8-fold greater in MRc40 than for CHO-K1, as measured using a clonogenic assay (Vallis and Wolf, 1996).

### High-performance liquid chromatography (HPLC)

Cells were grown to 75% confluency. Menadione (25 μM) was added to one flask of each cell line at the following times before cells were harvested: 24, 12, 6, 3, 2, 1 h and 20 min. Menadione was omitted from a control flask for each line. Cells were harvested, washed twice in phosphate-buffered saline (PBS), counted and a final cell suspension of $3 \times 10^6$ cells $ml^{-1}$ PBS prepared. These experiments were not continued beyond 24 h because by that time point CHO-K1 cells were beginning to die.

### Derivatization of samples

The method based on the sulphydryl-reactive agent, monobromobimane (mBBr), was used (Cotgreave and Moldéus, 1986). MBBr has high specificity and reactivity towards sulphydryls, with which it forms highly fluorescent adducts. The low molecular weight thiol–bimane adducts are suitable for chromatographic separation.

### Free (reduced) thiols

The following method was used to measure the intracellular concentrations of GSH and cysteine (CySH). A 100-μl aliquot of each whole cell suspension was transferred to an Eppendorf tube. Duplicate samples were prepared of both cell lines for each time point. PBS (10 μl) and 8 mM mBBr (100 μl) in 50 mM *N*-ethyl morpholine (pH 8.0) were added. MBBr is relatively insoluble and so was predissolved in acetonitrile (HPLC grade). Samples were stored at room temperature in darkness for 5 min and acidified to stop the reaction by the addition of 10 μl of 100% (w/v) trichloracetic acid. Precipitated protein was removed by centrifugation at 3000 × *g* for 10 min. Aliquots (100 μl) of the derivatized samples were transferred to glass vials before HPLC.

### Dithiothreitol reductions (total thiol)

In addition to measuring GSH and CySH, the following method allows recovery of GSH and CySH from their corresponding disulphide forms (GSSG and $CyS_2$) and from mixed protein thiols accessible to derivatization with monobromobimane. MBBr does not react directly with disulphides and so to render oxidized low molecular weight thiols and mixed protein thiols accessible to derivatization with mBBr, prederivatization reduction with dithiothreitol (DTT) was performed. Whole-cell suspensions (100 μl) were treated with 100 mM DTT, vortexed, stored for 30 min at room temperature and then derivatized by the addition of 20 mM mBBr in 50 mM *N*-ethyl morpholine (pH 8.0). The difference

**Figure 1** Effect of menadione on glutathione concentrration in CHO-K1 (○) and MRc40 (●) cells, (**A**) Total glutathione. (**B**) Free glutathione following the addition of 25 μM menadione. Each data point represents the mean and standard error of duplicate measurements. Where not shown, error bars fall within the area of the plot symbols. Results are from one representative experiment between total thiol concentration and free thiol concentration is a measure of the amount of low molecular weight thiol present in the disulphide form plus mixed protein thiols.

### HPLC separation of low molecular weight thiol–monobromobimane adducts

A Waters (Milford, MA, USA) Novapak steel column (3.9 × 150 mm) packed with 4 µm of octadodecyl silica reversed-phase material was used. The column was protected by a Waters Guard-Pak precolumn packed with the same material. The chromatographic system consisted of two model 410 pumps, an automated gradient controller, a Waters intelligent sampler processor model 710 (automatic injection system) and a data module (M730) for peak integration. A fluorescence detector (model 420) was used for peak detection. The elution solvent A was 10% (v/v) HPLC grade methanol and 0.25% (v/v) glacial acetic acid in distilled water (pH 3.9). Solvent B was 90% (v/v) methanol and 0.25% (v/v) glacial acetic acid in distilled water (pH 3.9). Buffers were degassed using a sonic bath. A water–methanol–acetic acid elution programme was used for chromatography as follows: 0–15 min, 100% buffer A; 15–25 min, 100% buffer B; 25–30 min 100% buffer A (column regeneration). Standard solutions of cysteine and GSH were prepared with PBS (pH 7.4) in the same way as test samples. To ensure consistency throughout the HPLC run, a vial containing standard thiol solution was placed in every fifth position on the sample processor.

**Figure 2** Effect of menadione on cysteine concentration in CHO-K1 (○) and MRc40 (●) cells. (**A**) Total and (**B**) free cysteine concentration following the addition of 25 µM menadione. Each data point represents the mean and standard error of duplicate measurements. Where not shown, error bars fall within the area of the plot symbols. Results are from one representative experiment

### Protein estimation

The cellular protein content was measured according to the method of Lowry et al (1951) using bovine serum albumin as standard.

### $^1H$ spin-echo NMR spectroscopy

Approximately $10^7$ cells were harvested, washed three times with $^2H_2O$/sodium chloride (0.154 M), suspended in 0.5 ml of $^2H_2O$/sodium chloride and transferred to a 5-mm NMR tube. NMR spectra were recorded using a Carr–Purcell–Meiboom–Gill sequence (90°–$t$–180°–$t$)$n$ with a delay time ($t$) of 60 ms and one repetition ($n$ = 1) of the pulse sequence per accumulation. A Buker 400 MHz spectrometer was used to record all spectra. Samples were maintained at room temperature during data collection and the data from 1024 complete pulse sequences were accumulated for each spectrum. The free induction decay (FID) was collected in 8 K of memory (acquisition time 0.64 seconds per scan) to which a 1.0-Hz line-broadening function applied, before zero filling and Fourier transformation. The 90° pulse was generated with a 15 µs pulse width. The water was eliminated from the spectrum by presaturation (55 dB) during relaxation delay (2 s). Once a satisfactory control spectrum had been obtained, menadione bisulphite was added. Menadione bisulphite was used in some NMR experiments because it is more soluble in water than menadione. The ethanol required to dissolve menadione itself interferes with NMR spectra.

### RESULTS

HPLC was used to measure total and free low molecular weight thiols in CHO-K1 and MRc40 (Figures 1 and 2). The concentration of total glutathione in CHO-K1 and MRc40 was 6.0 and 12.1 nmol mg$^{-1}$ protein respectively (Figure 1A). Following addition of menadione, the level of glutathione fell rapidly in both cell lines. However, depletion of glutathione was more profound and its recovery delayed for longer in CHO-K1 compared with MRc40. In MRc40, glutathione concentration had returned to the control level by 6 h. In CHO-K1, this took 24 h. The rise in glutathione concentration in MRc40 exceeded the pretreatment value. The maximum concentration of total glutathione in MRc40 was 26.3 nmol mg$^{-1}$ protein, at 12 h. This is a 2.2-fold increase in glutathione compared with the resting state. The level of glutathione in CHO-K1 did not rise significantly above the pretreatment level during recovery. By 12 h after the addition of menadione, the concentration of total glutathione in MRc40 cells was 9.7-fold greater than in CHO-K1.

A similar pattern of initial depletion followed by recovery is also seen with intracellular cysteine (Figure 2). Resting concentration of total cysteine in CHO-K1 and MRc40 was 2.5 and 7.9 nmol mg$^{-1}$

© Cancer Research Campaign 1997

**Figure 3** Effect of menadione on the relative concentrations of total (■) and free (□) thiols in CHO cell lines. (**A**) Total and free glutathione in CHO-K1. (**B**) Total and free glutathione MRc40. (**C**) Total and free cysteine in CHO-K1. (**D**) Total and free cysteine in MRc40

protein respectively (Figure 2A). This represents a 3.2-fold elevation of cysteine in MRc40 compared with CHO-K1. The level of cysteine during the recovery phase is greater for MRc40 than for CHO-K1. The maximum concentration of total cysteine in MRc40 was 10.5 nmol/mg$^{-1}$ protein (at 24 h). This is a 1.3-fold increase in cysteine compared with cells in the resting state and a fourfold increase in cysteine compared with CHO-K1 at the same time point.

The difference between total and free (reduced) thiol concentration values is a measure of the thiol present in the oxidized (disulphide) form plus oxidized protein thiols. In Figure 3, the data from the experiment shown in Figures 1 and 2 is replotted to allow comparison of the concentrations of free and total glutathione and cysteine. In the case of glutathione, taking error bars into account, the free and total thiol concentrations are almost identical and this is also true following the addition of menadione. This demonstrates that the redox status of glutathione lies strongly in favour of the reduced form. This is in agreement with others who have found that for cultured cells almost all glutathione is in the reduced form (Cotgreave and Moldéus, 1986). In contrast, Figure 3 shows that the concentration of total cysteine significantly exceeds the concentration of free thiol. The redox status of cysteine lies more in favour of the disulphide species. This is true of both MRc40 and CHO-K1.

The $^{1}H$ spin-echo NMR spectra of CHO-K1 and MRc40 cells are shown in Figure 4. There were more cells in the CHO-K1 ($3 \times 10^7$) than in the MRc40 sample ($1.5 \times 10^7$). However, the spectral patterns obtained for the two cell lines were similar and the resonances caused by glutathione were identifiable in both. The amount of NMR-visible GSH is similar in the two cell lines. In both cell types, approximately 95% of the glutathione is in the reduced state. Addition of menadione to CHO-K1 cells is followed by several changes in the NMR spectrum (Figure 5). Two control spectra were obtained at an interval of 1 h, showing that there is no significant change in the spectra until drug is added. Following the addition of menadione, the peaks assigned to glutathione diminish from 30 min. This is particularly obvious for the g1 peak (caused by the glycine methyl group). At 12 h, the resonances resulting from glutathione are barely detectable. These NMR experiments necessitated maintaining cells in PBS at room temperature overnight. A control experiment showed that the spectra obtained at the start and at the end of such experiments (i.e. at 12 h) were not significantly different (data not shown). Cells maintain their viability in these circumstances and so changes in the spectra are attributed to the effects of menadione.

Incubation of CHO-K1 cells with 100 μM menadione bisulphite caused rapid depletion of NMR-visible glutathione. This is shown in Figure 6, in which g1 peak height is plotted against time. In contrast, incubation of MRc40 cells with 100 μM and 500 μM menadione bisulphite did not cause a change in intracellular glutathione. NMR spectroscopy allows observation of menadione bisulphite in the cell suspensions. In both CHO-K1 and MRc40, we observed depletion of menadione. This is demonstrated in Figure 7 in which the height of the peak assigned to menadione is plotted against time. Depletion was more rapid in MRc40 than in CHO-K1, suggesting more efficient metabolism of menadione in

© Cancer Research Campaign 1997

**Figure 4** Glutathione content determined by NMR. (**A**) CHO-K1. (**B**) MRc40 cells. The four resonances assigned to glutathione are indicated as g1–g4. The number of cells were 3 × $10^7$ and 1.5 × $10^7$ for CHO-K1 and MRc40 respectively

**Figure 5** Effect of menadione on glutathione concentration in CHO-K1. NMR spectra obtained at 0.5, 1 and 12 h after the addition of 25 μM menadione to CHO-K1 cells. Control spectra were taken 1 h apart the resistant cells. It is significant that, in MRc40, menadione is metabolized without concomitant change in the NMR-visible pool of GSH. In none of the NMR spectroscopy data is there evidence to support the formation of a glutathione–menadione conjugate or significant amounts of GSSG.

### DISCUSSION

In a previous report, we described the isolation and characterization of a menadione-resistant cell line, MRc40 (Vallis and Wolf, 1996). Menadione resistance was associated with cross-resistance to some other oxidants and with changes in the pattern of gene expression. Of particular interest were changes in the expression of genes, such as haem oxygenase, induced by transient exposure of the cells to oxidative stress. In addition, elevation of glutathione and cysteine was observed in the resistant compared with the wild-type cells. Two other groups have measured the glutathione content of menadione-resistant cell lines. Martins and Meneghini (1990) found a 1.5-fold increase in glutathione in a menadione-resistant variant of V79 Chinese hamster cells. Ngo and Nutter (1994) made a menadione-resistant subline of the human breast carcinoma line, MCF-7. There was no alteration in GSH content. The concentration of cysteine was not measured in either of these cell lines, and the ability of cells to synthesize glutathione under conditions of oxidative stress was not investigated.

Measurement of GSH by HPLC demonstrated that the cell line, MRc40, has a greater capacity for resynthesis of GSH than CHO-K1. GSH synthesis occurs as a result of the sequential action of γ-glutamylcysteine synthetase (γ-GCS) and glutathione synthetase. γ-GCS is rate limiting and a negative feedback mechanism operates in which glutathione inhibits γ-GCS and so inhibits its own synthesis (Richman and Meister, 1975). The cellular regulation of glutathione plays an important role in protection from various oxidative insults. For example, Moore et al (1989) found that a strain of *E. coli* enriched in the genes for γ-GCS and glutathione synthetase was more resistant to cell killing by γ-irradiation than the corresponding wild strain. In bacteria, the concentration of glutathione varies markedly with the phase of growth, being high

© Cancer Research Campaign 1997

**Figure 6** Effect of menadione on glutathione content in CHO cell lines. G1 peaks from successive spectra (obtained 30 min apart) are plotted against time. CHO-K1 following 100 µM menadione bisulphite (●). MRc40 cells following 100 µM menadione bisulphite (■). MRc40 cells following 500 µM menadione bisulphite (□)

**Figure 7** Metabolism of menadione in CHO cell lines determined by NMR. Height of peak attributable to menadione bisulphite plotted against time. CHO-K1 (●). MRc40 (□). The peak attributable to menadione bisulphite was scanned at 20 min intervals following the addition of 100 µM menadione during stationary phase and low during logarithmic growth. Survival following radiation did not vary with the phase of growth of wild-strain bacteria, suggesting that the intracellular concentration of GSH at the time of irradiation is not an important factor in the determination of survival. The relative radioresistance of the gene-enriched strain was associated with an increased capacity to synthesize glutathione immediately after irradiation rather than the absolute concentration of glutathione per se. Therefore, the ability to synthesize glutathione appears to be at least as important as concentration.

Glutathione synthesis is stimulated by cysteine, one of its three constituent amino acids (Bannai and Tateishi, 1986). Cysteine oxidizes to cystine in extracellular fluid, whereas cystine is rapidly reduced to cysteine when it enters the cells. An anionic amino acid transport system that is highly specific for cystine and glutamate has been described in various cells and has been designated $\chi_c^-$ (Bannai, 1986). Deneke (1992) studied bovine pulmonary artery endothelial cells and found that agents that cause oxidative stress resulted in the induction of cystine transport. Similar observations were made when human umbilical vein endothelial cells were exposed to hydrogen peroxide (Miura et al, 1992a). It seems likely that up-regulation of cystine transport is an important adaptive response to oxidative stress in vivo. The HPLC data presented in this paper show that, after addition of menadione, cysteine concentration falls initially but then rises above normal levels. It is interesting to speculate that one of the mechanisms by which the MRc40 line has acquired resistance to menadione is through the improved efficiency of cystine uptake from the culture medium and that this in turn allows rapid replenishment of GSH.

The HPLC data presented in Figure 3 show that the redox status of glutathione lies in favour of the reduced form, whereas that of cysteine lies more in favour of the disulphide species. This is true of a variety of different cell types that have been tested (Cotgreave and Moldéus, 1986). Others have reported the production of substantial amounts of GSSG following addition of menadione to isolated hepatocytes (Ross et al, 1985). We did not observe the formation of GSSG in the HPLC experiments, since there was no significant difference between total and free thiol concentrations. As long as there is adequate glutathione reductase activity, GSSG produced during free radical scavenging is rapidly converted to GSH and we assume that that is why it was not detectable. It is interesting that the HPLC and NMR spectroscopy data from the present study support one another. In neither was there evidence for the formation of significant amounts of GSSG. If glutathione is in the reduced state, the NMR resonance, g2, is negative and larger than g4, but in the oxidized state it is smaller. There was no NMR evidence for the formation of substantial amounts of GSSG.

Although HPLC provides accurate quantitative data of intracellular thiol status, NMR has the advantage that biochemical changes can be studied without prior disruption of the cell. NMR has been used by a number of investigators to compare the physiological characteristics of drug-resistant cell lines with the parental lines from which they were isolated. For example, Jiang et al (1993) found significantly lower taurine content in a drug-resistant T-lymphoid leukaemia cell line compared with its drug-sensitive parental counterpart. De Jong et al (1991) used $^{31}$P- and $^{1}$H-NMR spectroscopy to measure phospholipid metabolism in a doxorubicin-resistant human small-cell lung carcinoma cell line and the corresponding doxorubicin-sensitive parental cell line. This is the first report, to our knowledge, in which NMR has been used to study glutathione homeostasis in relation to drug resistance. Following the addition of menadione, the NMR-visible GSH falls in CHO-K1 cells (Figures 5 and 6), and there is no subsequent recovery over 12 h (Figure 5). During these experiments, cells are suspended in PBS and so uptake of cystine from the suspending medium and subsequent recovery of GSH would be limited. Garner et al (1997) carried out similar experiments using NMR spectroscopy to follow the response of erythrocytes to the treatment of the oxidizing agent, vanadate. Pretreating the cells with nitrofurantoin, which blocks glutathione reductase, induces a profound depletion of GSH. As the cells were metabolically dormant, no recovery of GSH was observed. The MRc40 cells were better able to maintain the pool of intracellular GSH during oxidative stress than CHO-K1 (Figure 6). Using NMR, we did not observe an initial fall in GSH in MRc40 after the addition of menadione, as we had done using HPLC. This apparently contradictory result may result from differences in GSH homeostasis in intact cells compared with cells disrupted during the measurement of GSH. Another possible explanation is that, during the NMR experiments, cells are relatively hypoxic since a large number of cells are packed into a relatively small volume. Since molecular oxygen is required for redox cycling by menadione, the level of oxidative stress in the NMR model may have been limited and the MRc40 cells, therefore, better able to maintain the intracellular level of GSH.

Menadione is capable of covalent binding to protein (Di Monte et al, 1984). Binding of menadione to protein renders it NMR invisible, since only small molecules are discernible using the particular NMR technique described here. We therefore hypothesize that the apparent loss of menadione shown in Figure 7 is caused by covalent binding of drug to protein. This occurs to a greater degree in the resistant cells compared with the sensitive cells. This raises the possibility that the resistant cells are richer in protein-SH groups than wild-type cells, and that enhanced drug binding by protein is one of the adaptive mechanisms leading to resistance in the MRc40 cell line.

Others have reported the identification, by ESR spectroscopy, of semiquinone and hydroquinone radical formation during the intracellular metabolism of menadione (Miura et al, 1992*b*). There is little evidence in the NMR spectra to support the efficient formation of either a menadione hydroquinone or semiquinone via mitochondrial oxidation. If a hydroquinone or semiquinone was produced, it would not itself be NMR visible. However, the associated paramagnetism would significantly broaden the resonances in the spectra and this is not observed. Generation of low-concentration radicals would affect T2, altering the shape of the FID and the strength of the lock signal. Neither is observed. This is another indication that redox cycling of menadione is absent or minimal in the NMR model.

The formation of a GSH–menadione conjugate has been observed by others (Nickerson et al, 1963). However, we saw no resonances that could be ascribed to such a conjugate in the NMR spectra of CHO whole cells. The formation of the menadione–GSH conjugate is sensitive to the relative concentrations of menadione and glutathione (Nickerson et al, 1963). It would appear that in the model used here the conditions were not conducive to the formation of a conjugate.

The measurement of cellular thiols using NMR spectroscopy and HPLC indicates that glutathione metabolism is profoundly altered in menadione-resistant cells, and it is likely that this alteration contributes to the resistant phenotype. Quinones such as menadione produce a flux of free radicals during redox cycling. Glutathione is important in the detoxification of free radicals. Thus, the elevated resting concentration of GSH and increased capacity for GSH replenishment seen in MRc40 would serve to protect these cells from free radical damage. The use of NMR spectroscopy to study glutathione homeostasis serves as a useful tool with which to elucidate further the role of this important molecule in drug resistance.

### ABBREVIATIONS

CHO, Chinese hamster ovary; HPLC, high-performance liquid chromatography; NMR, nuclear magnetic resonance; GSH, reduced glutathione; GSSG, disulphide glutathione; CySH, cysteine; $CyS_2$, cystine; $H_2O_2$, hydrogen peroxide; γ-GCS, gamma glutamylcysteine synthetase; PBS, phosphate-buffered saline; mBBr, monobromobimane; ESR, electron spin resonance.

### ACKNOWLEDGEMENT

MG wishes to express his gratitude for financial assistance from the Department of Home and Health, Scotland.

### REFERENCES

Al-Kabban M, Watson ID, Stewart MJ, Reglinski J, Smith WE and Suckling CJ (1988) The use of $^1$H spin echo NMR and HPLC to confirm doxorubicin induced depletion of glutathione in the intact HeLa cell. *Br J Cancer* **57**: 553–558

Bannai S (1986) Exchange of cystine and glutamate across plasma membrane of human fibroblasts. *J Biol Chem* **261**: 2256–2263

Bannai S and Tateishi N (1986) Role of membrane transport in metabolism and function of glutathione in mammals. *J Membr Biol* **89**: 1–8

Chlebowski RT, Akman SA and Block JB (1985) Vitamin K in the treatment of cancer. *Cancer Treat Rev* **12**: 49–63

Cotgreave IA and Moldéus P (1986) Methodologies for the application of monobromobimane to the simultaneous analysis of soluble and protein thiol components of biological systems. *J Biochem Biophys Methods* **13**: 231–249

Deeley TJ (1962) A clinical trial of synkavit in the treatment of carcinoma of the bronchus. *Br J Cancer* **16**: 387–389

De Jong S, Mulder NH, De Vries Ege E and Robillard GT (1991) NMR spectroscopy analysis of phosphorus metabolites and the effect of adriamycin on these metabolite levels in an adriamycin-sensitive and -resistant human small cell lung carcinoma cell line. *Br J Cancer* **63**: 205–212

Deneke SM (1992) Induction of cystine transport in bovine pulmonary artery endothelial cells by sodium arsenite. *Biochim Biophys Acta* **1109**: 127–131

Dethmers JK and Meister A (1981) Glutathione export by human lymphoid cells: depletion of glutathione by inhibition of its synthesis decreases export and increases sensitivity to irradiation. *Proc Natl Acad Sci USA* **78**: 7492–7496

Di Monte D, Bellomo G, Thor H, Nicotera P and Orrenius S (1984) Menadione-induced cytotoxicity is associated with protein thiol oxidation and alteration in intracellular $Ca^{2+}$ homeostasis. *Arch Biochem Biophys* **235**: 343–350

Garner M, Reglinski J, Smith WE, McMurray J, Abdullah I and Wilson R (1997) A $^1$H spin echo and $^{51}$V NMR study of the interaction of vanadate with intact erythrocytes. *J Biol Inorg Chem* **2**: 235–241

Greenberg JT, Monach P, Chou JH, Josephy PD and Demple B (1990) Positive control of a global antioxidant defense regulon activated by superoxide-generating agents in *Escherichia coli*. *Proc Natl Acad Sci USA* **87**: 6181–6185

Jiang XR, Yang M, Morris CJ, Newland AC, Naughton DP, Blake DR, Zhang Z and Grootveld MC (1993) High field proton NMR investigations of the metabolic profiles of multidrug-sensitive and -resistant leukaemic cell lines: evidence for diminished taurine levels in multidrug-resistant cells. *Free Rad Res Commun* **19**: 355–369

Kramer RA, Zakher J and Kim G (1988) Role of the glutathione redox cycle in acquired and de novo multidrug resistance. *Science* **241**: 694–697

Livesey JC, Golden RN, Shankland EG, Grunbaum Z, Wyman M and Krohn KA (1992) Magnetic resonance spectroscopic measurement of cellular thiol reduction–oxidation state. *Int J Radiat Oncol Biol Phys* **22**: 755–757

Lowry OH, Rosebrough NJ, Farr AL and Randall RJ (1951) Protein measurements with the folin phenol reagent. *J Biol Chem* **193**: 265–275

McKay CNN, Brown DH, Reglinski J, Smith WE, Capell HA and Sturrock RD (1986) Changes in glutathione in intact erythrocytes during incubation with penicillamine as detected by $^1$H spin-echo NMR spectroscopy. *Biochim Biophys Acta* **888**: 30–35

Margolin KA, Akman SA, Leong LA, Morgan RJ, Somlo G, Raschko JW, Ahn C and Doroshow JH (1995) Phase I study of mitomycin C and menadione in advanced solid tumours. *Cancer Chemother Pharmacol* **36**: 293–298

Martins EAL and Meneghini R (1990) DNA damage and lethal effects of hydrogen peroxide and menadione in Chinese hamster cells: distinct mechanisms are involved. *Free Rad Biol Med* **8**: 433–440

Mason RP (1982) Free radical intermediates in the metabolism of toxic chemicals. In *Free Radicals in Biology*, Pryor WA (ed.) pp. 165–174. Academic Press: New York

Miura K, Ishii T, Sugita Y and Bannai S (1992*a*) Cystine uptake and glutathione level in endothelial cells exposed to oxidative stress. *Am J Physiol* **262**: C50–C58

Miura T, Muraoka S and Ogiso T (1992*b*) Generation of semiquinone and oxygen radicals by the reaction of menadione with reduced glutathione at various pH. *Chem Pharm Bull* **40**: 709–712

Moore WR, Anderson ME, Meister A, Murata K and Kimura A (1989) Increased capacity for glutathione synthesis enhances resistance to radiation in

 © Cancer Research Campaign 1997

*Escherichia coli*: a possible model for mammalian cell protection. *Proc Natl Acad Sci USA* **86**: 1461–1464

Ngo EO and Nutter LM (1994) Status of glutathione and glutathione-metabolizing enzymes in menadione-resistant human cancer cells. *Biochem Pharmacol* **47**: 421–424

Nickerson WJ, Falcone G and Strauss G (1963) Studies on quinone thioethers. I Mechanism of formation and properties of thiodione. *Biochemistry* **2**: 537–543

Richman PG and Meister A (1975) Regulation of γ-glutamyl-cysteine synthetase by nonallosteric feedback inhibition by glutathione. *J Biol Chem* **250**: 1422–1426

Ross D, Thor H, Orrenius S and Moldéus P (1985) Interaction of menadione (2-methyl-1,4-naphthoquinone) with glutathione. *Chem Biol Interact* **55**: 177–184

Russo A, Mitchell JB, Finkelstein E, De Graff WG, Spiro IJ and Gamson J (1985) The effects of cellular glutathione elevation on the oxygen enhancement ratio. *Radiat Res* **103**: 232–239

Thor H, Smith MT, Hartzell P, Bellomo G, Jewell SA and Orrenius S (1982) The metabolism of menadione (2-methyl-1,4-naphthoquinone) by isolated hepatocytes. A study of the implications of oxidative stress in intact cells. *J Biol Chem* **257**: 12419–12425

Vallis KA and Wolf CR (1996) Relationship between the adaptive response to oxidants and stable menadione-resistance in Chinese hamster ovary cell lines. *Carcinogenesis* **17**: 649–654

Vos O and Roos-Verhey WSD (1988) Radioprotection by glutathione esters and cysteamine in normal and glutathione-depleted mammalian cells. *Int J Radiat Biol* **53**: 273–281

Wolf CR, Lewis AD, Carmichael J, Adams DJ, Allan SG and Ansell DJ (1987) The role of glutathione in determining the response of normal and tumour cells to anticancer drugs. *Biochem Soc Trans* **15**: 728–730

© Cancer Research Campaign 1997


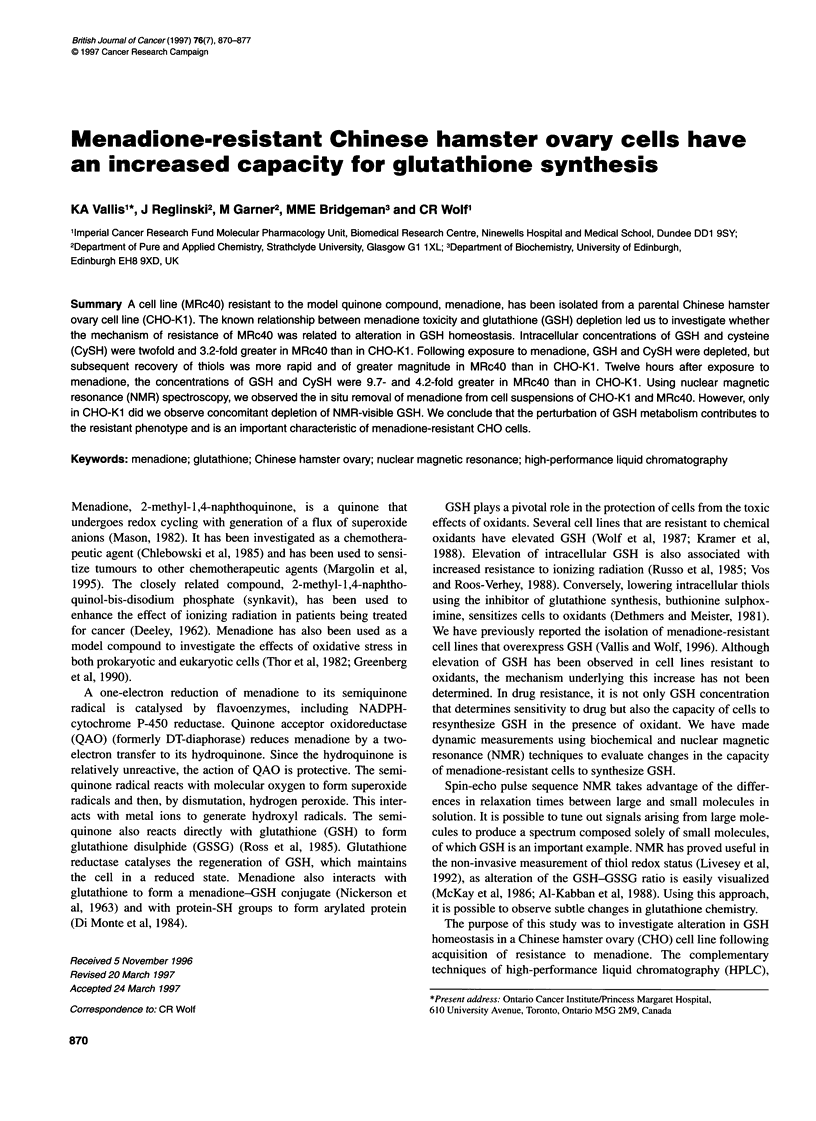

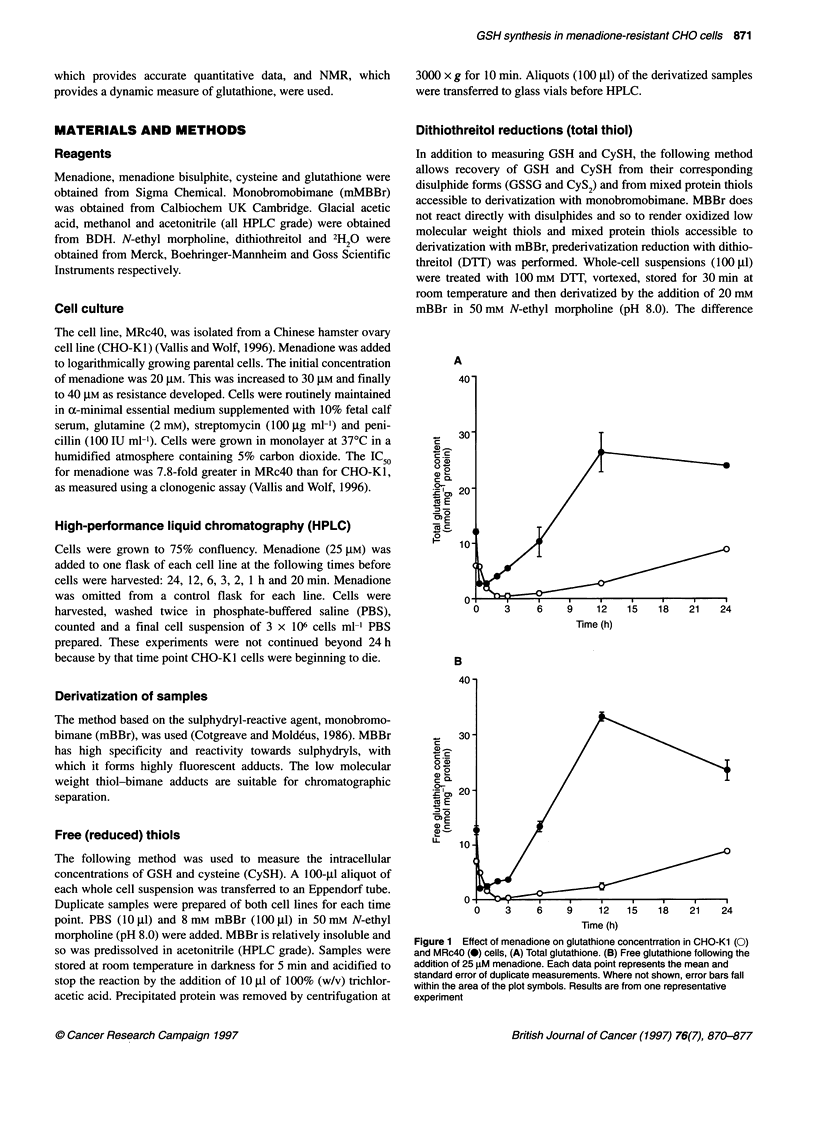

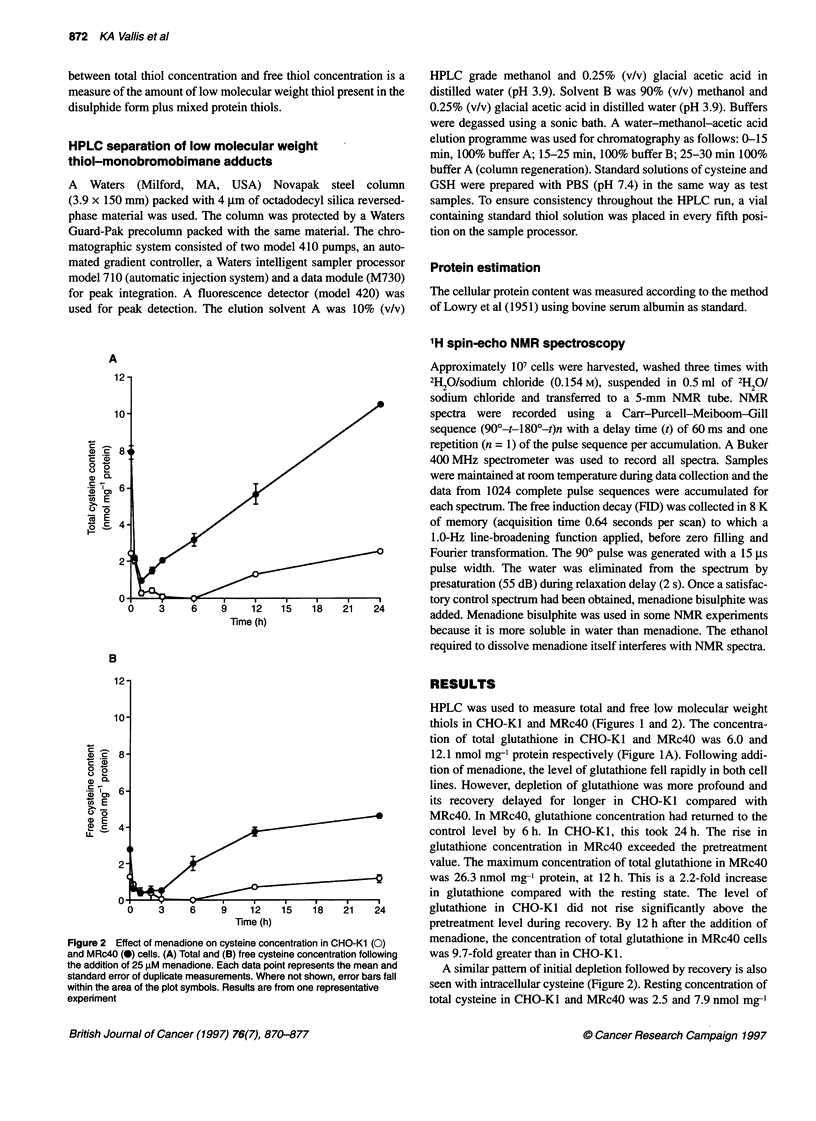

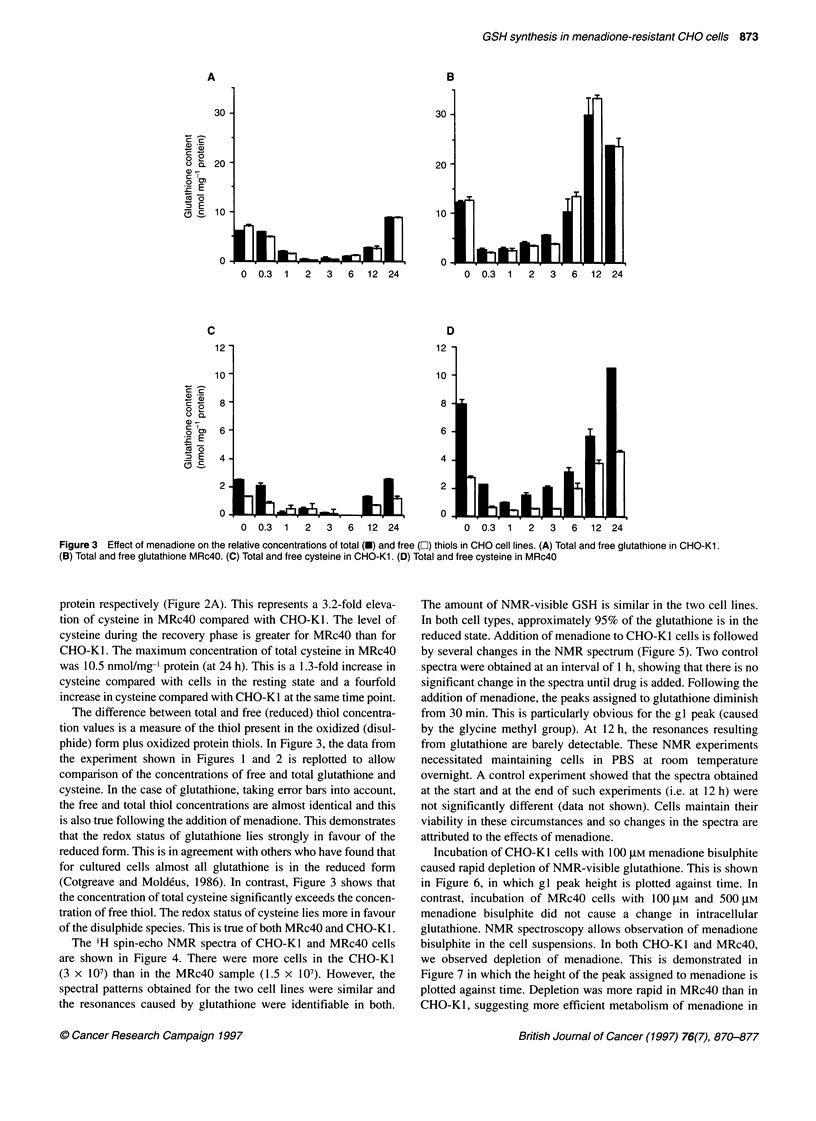

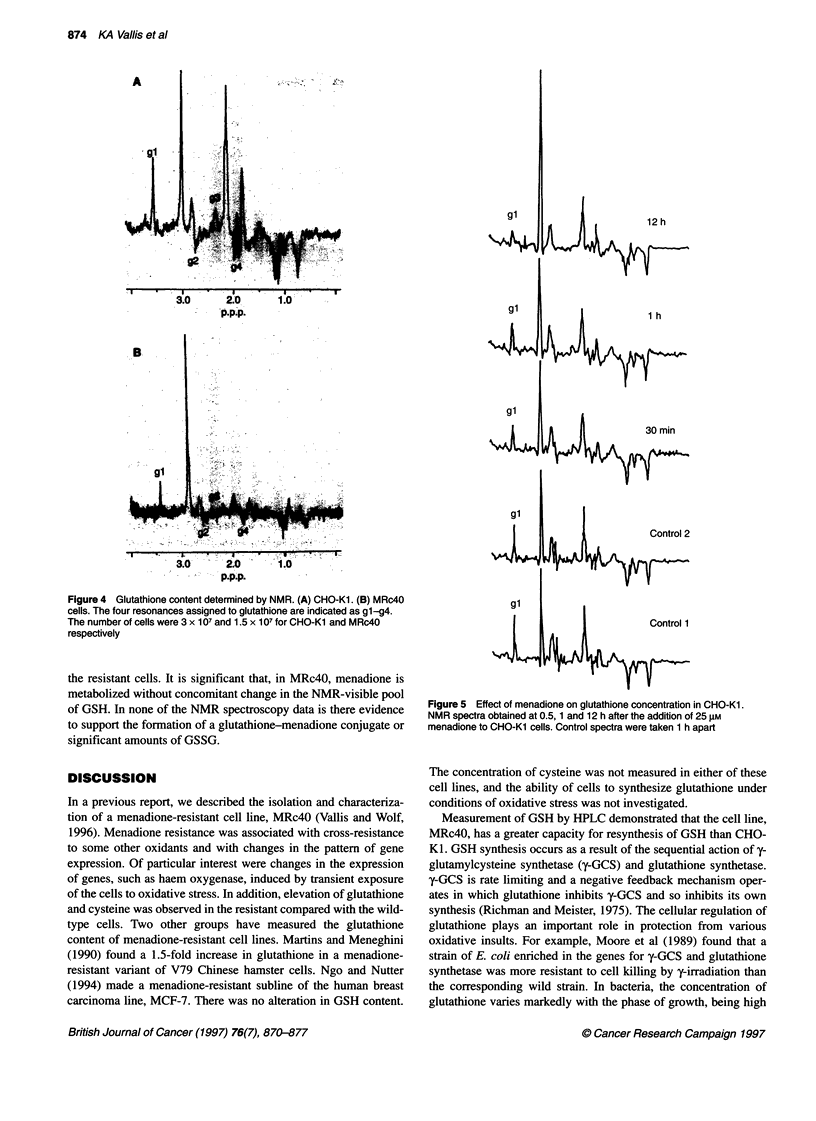

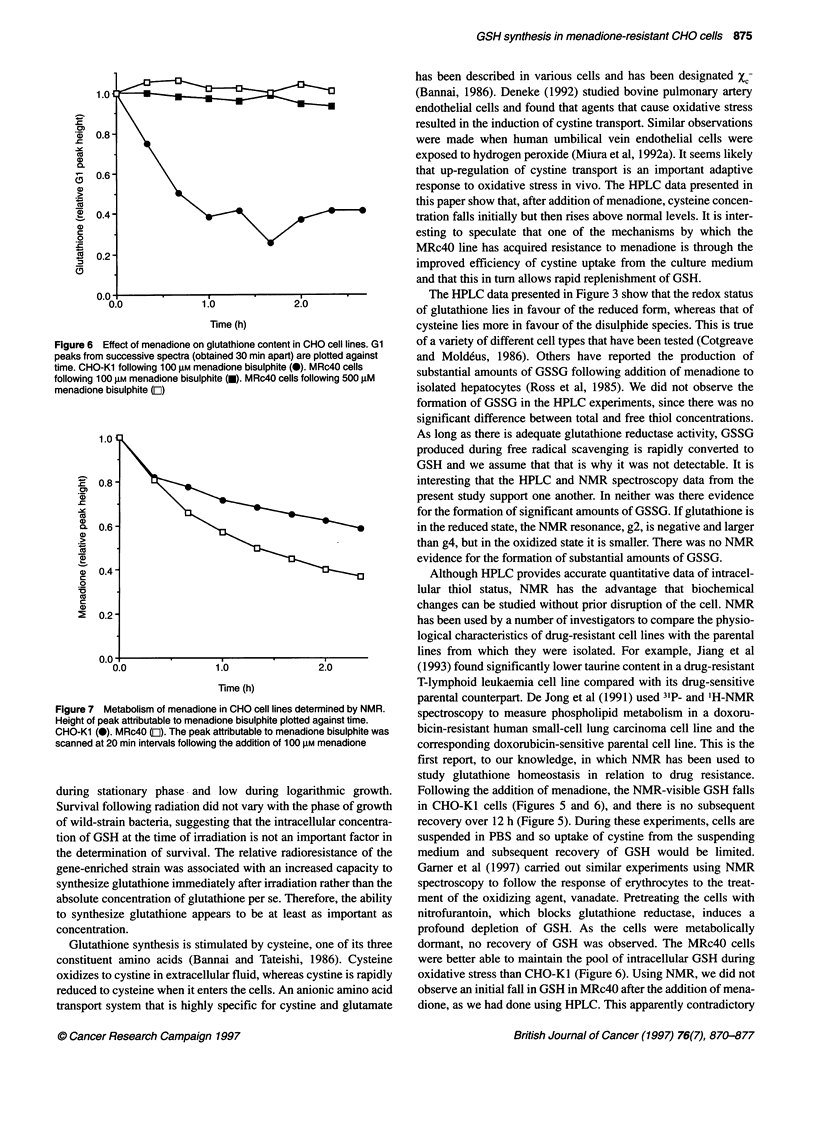

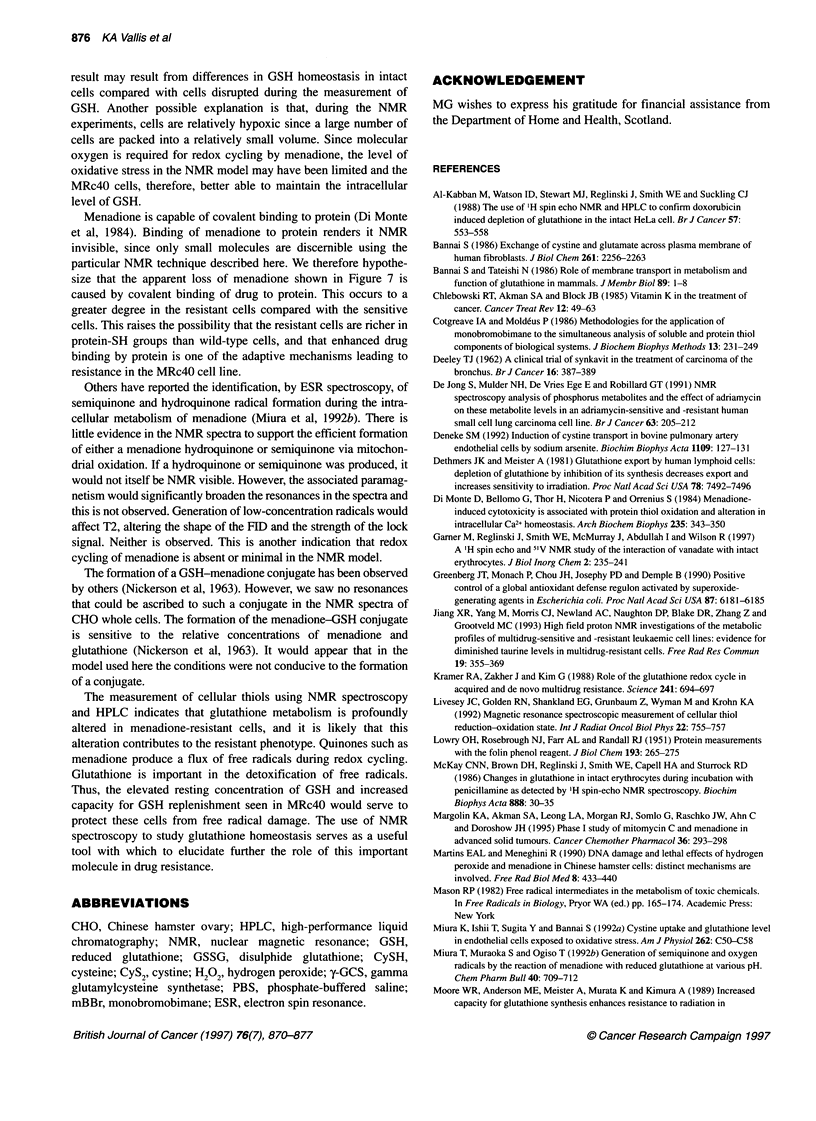

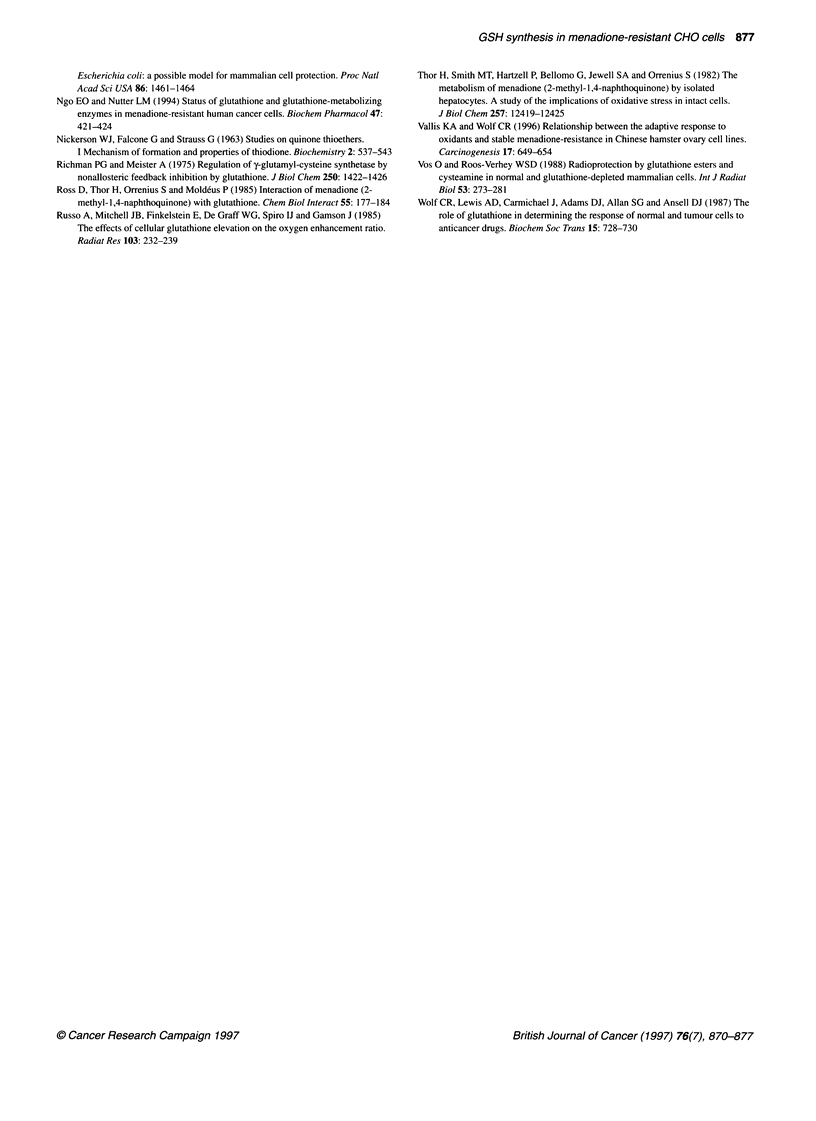


## References

[OCR_00804] Bannai S. (1986). Exchange of cystine and glutamate across plasma membrane of human fibroblasts.. J Biol Chem.

[OCR_00808] Bannai S., Tateishi N. (1986). Role of membrane transport in metabolism and function of glutathione in mammals.. J Membr Biol.

[OCR_00812] Chlebowski R. T., Akman S. A., Block J. B. (1985). Vitamin K in the treatment of cancer.. Cancer Treat Rev.

[OCR_00816] Cotgreave I. A., Moldéus P. (1986). Methodologies for the application of monobromobimane to the simultaneous analysis of soluble and protein thiol components of biological systems.. J Biochem Biophys Methods.

[OCR_00821] DEELEY T. J. (1962). A clinical trial of synkavit in the treatment of carcinoma of the bronchus.. Br J Cancer.

[OCR_00831] Deneke S. M. (1992). Induction of cystine transport in bovine pulmonary artery endothelial cells by sodium arsenite.. Biochim Biophys Acta.

[OCR_00835] Dethmers J. K., Meister A. (1981). Glutathione export by human lymphoid cells: depletion of glutathione by inhibition of its synthesis decreases export and increases sensitivity to irradiation.. Proc Natl Acad Sci U S A.

[OCR_00839] Di Monte D., Bellomo G., Thor H., Nicotera P., Orrenius S. (1984). Menadione-induced cytotoxicity is associated with protein thiol oxidation and alteration in intracellular Ca2+ homeostasis.. Arch Biochem Biophys.

[OCR_00849] Greenberg J. T., Monach P., Chou J. H., Josephy P. D., Demple B. (1990). Positive control of a global antioxidant defense regulon activated by superoxide-generating agents in Escherichia coli.. Proc Natl Acad Sci U S A.

[OCR_00854] Jiang X. R., Yang M., Morris C. J., Newland A. C., Naughton D. P., Blake D. R., Zhang Z., Grootveld M. C. (1993). High field proton NMR investigations of the metabolic profiles of multidrug-sensitive and -resistant leukaemic cell lines: evidence for diminished taurine levels in multidrug-resistant cells.. Free Radic Res Commun.

[OCR_00862] Kramer R. A., Zakher J., Kim G. (1988). Role of the glutathione redox cycle in acquired and de novo multidrug resistance.. Science.

[OCR_00871] LOWRY O. H., ROSEBROUGH N. J., FARR A. L., RANDALL R. J. (1951). Protein measurement with the Folin phenol reagent.. J Biol Chem.

[OCR_00866] Livesey J. C., Golden R. N., Shankland E. G., Grunbaum Z., Wyman M., Krohn K. A. (1992). Magnetic resonance spectroscopic measurement of cellular thiol reduction-oxidation state.. Int J Radiat Oncol Biol Phys.

[OCR_00881] Margolin K. A., Akman S. A., Leong L. A., Morgan R. J., Somlo G., Raschko J. W., Ahn C., Doroshow J. H. (1995). Phase I study of mitomycin C and menadione in advanced solid tumors.. Cancer Chemother Pharmacol.

[OCR_00886] Martins E. A., Meneghini R. (1990). DNA damage and lethal effects of hydrogen peroxide and menadione in Chinese hamster cells: distinct mechanisms are involved.. Free Radic Biol Med.

[OCR_00875] McKay C. N., Brown D. H., Reglinski J., Smith W. E., Capell H. A., Sturrock R. D. (1986). Changes in glutathione in intact erythrocytes during incubation with penicillamine as detected by 1H spin-echo NMR spectroscopy.. Biochim Biophys Acta.

[OCR_00896] Miura K., Ishii T., Sugita Y., Bannai S. (1992). Cystine uptake and glutathione level in endothelial cells exposed to oxidative stress.. Am J Physiol.

[OCR_00904] Moore W. R., Anderson M. E., Meister A., Murata K., Kimura A. (1989). Increased capacity for glutathione synthesis enhances resistance to radiation in Escherichia coli: a possible model for mammalian cell protection.. Proc Natl Acad Sci U S A.

[OCR_00920] NICKERSON W. J., FALCONE G., STRAUSS G. (1963). STUDIES ON QUINONE-THIOETHERS. I. MECHANISM OF FORMATION AND PROPERTIES OF THIODIONE.. Biochemistry.

[OCR_00915] Ngo E. O., Nutter L. M. (1994). Status of glutathione and glutathione-metabolizing enzymes in menadione-resistant human cancer cells.. Biochem Pharmacol.

[OCR_00923] Richman P. G., Meister A. (1975). Regulation of gamma-glutamyl-cysteine synthetase by nonallosteric feedback inhibition by glutathione.. J Biol Chem.

[OCR_00926] Ross D., Thor H., Orrenius S., Moldeus P. (1985). Interaction of menadione (2-methyl-1,4-naphthoquinone) with glutathione.. Chem Biol Interact.

[OCR_00929] Russo A., Mitchell J. B., Finkelstein E., DeGraff W. G., Spiro I. J., Gamson J. (1985). The effects of cellular glutathione elevation on the oxygen enhancement ratio.. Radiat Res.

[OCR_00934] Thor H., Smith M. T., Hartzell P., Bellomo G., Jewell S. A., Orrenius S. (1982). The metabolism of menadione (2-methyl-1,4-naphthoquinone) by isolated hepatocytes. A study of the implications of oxidative stress in intact cells.. J Biol Chem.

[OCR_00941] Vallis K. A., Wolf C. R. (1996). Relationship between the adaptive response to oxidants and stable menadione-resistance in Chinese hamster ovary cell lines.. Carcinogenesis.

[OCR_00946] Vos O., Roos-Verhey W. S. (1988). Radioprotection by glutathione esters and cysteamine in normal and glutathione-depleted mammalian cells.. Int J Radiat Biol Relat Stud Phys Chem Med.

[OCR_00951] Wolf C. R., Lewis A. D., Carmichael J., Adams D. J., Allan S. G., Ansell D. J. (1987). The role of glutathione in determining the response of normal and tumor cells to anticancer drugs.. Biochem Soc Trans.

[OCR_00798] al-Kabban M., Watson I. D., Stewart M. J., Reglinski J., Smith W. E., Suckling C. J. (1988). The use of 1H spin echo NMR and HPLC to confirm doxorubicin induced depletion of glutathione in the intact HeLa cell.. Br J Cancer.

[OCR_00825] de Jong S., Mulder N. H., de Vries E. G., Robillard G. T. (1991). NMR spectroscopy analysis of phosphorus metabolites and the effect of adriamycin on these metabolite levels in an adriamycin-sensitive and -resistant human small cell lung carcinoma cell line.. Br J Cancer.

